# Supplementary material for: Yiai Fuzheng decoction inhibits triple-negative breast cancer by remodeling the immune microenvironment
Source: Front Immunol. 2025 Sep 30;16:1615631. doi: 10.3389/fimmu.2025.1615631 (PMC12518410; doi:10.3389/fimmu.2025.1615631)
Supplement: Supplementary file 2 [file DataSheet1.zip › Supplementary File 2/Treatment/C_vs_D/07_Pathway_Analysis/Pathway_Analysis/Pathway_Barplot.pdf]

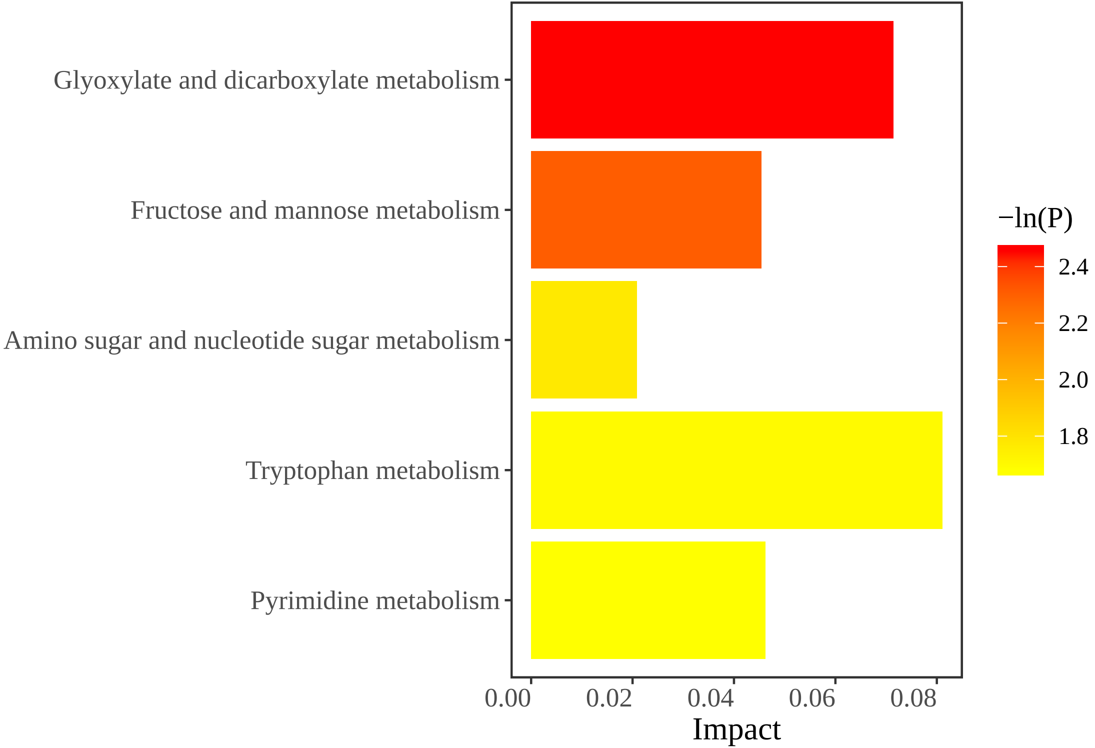

Glyoxylate and dicarboxylate metabolism
Fructose and mannose metabolism
Amino sugar and nucleotide sugar metabolism
Tryptophan metabolism
Pyrimidine metabolism
0.00
0.02
0.04
0.06
0.08
Impact
−ln(P)
2.4
2.2
2.0
1.8
